# Supplementary material for: A weighting method for simultaneous adjustment for confounding and joint exposure-outcome misclassifications
Source: Stat Methods Med Res. 2020 Sep 30;30(2):473–87. doi: 10.1177/0962280220960172 (PMC8008432; doi:10.1177/0962280220960172)
Supplement: sj-pdf-1-smm-10.1177_0962280220960172 - Supplemental material for A weighting method for simultaneous adjustment for confounding and joint exposure-outcome misclassifications [file sj-pdf-1-smm-10.1177_0962280220960172.pdf]

Supplementary Web Appendix: A validation subset weighting method for outcome and exposure misclassification and confounding

Bas B.L. Penning de Vries[1] and Maarten van Smeden[1], and Rolf H.H. Groenwold[1,2]

[1] Department of Clinical Epidemiology, Leiden University Medical Center, The Netherlands
[2] Department of Biomedical Data Sciences, Leiden University Medical Center, The Netherlands

Corresponding author:
Bas B.L. Penning de Vries, Department of Clinical Epidemiology, Leiden University Medical Center, PO Box 9600, 2300 RC, The Netherlands
Email: B.B.L.Penning_de_Vries@lumc.nl

Appendix I

Suppose A, B, Y and Z are random variables that take values in $\{0, 1\}$.

Theorem S.1. *For any a, l, let*

$$\varphi(a,l) = \frac{\varphi^*(a,l)}{\mathbb{E}[\varphi^*(A,L)|A=a]} \text{ and } \varphi^*(a,l) = \frac{1}{\Pr(A=a|L=l)}.$$

If $Y(A) = Y$ (consistency), $(Y(0), Y(1)) \perp\!\!\!\perp A|L = l$ (conditional exchangeability), $\Pr(A = a) > 0$ and $\Pr(A = a|L = l) > 0$ (positivity) for all a and every l in the support of L, then

$$\mathbb{E}[Y(a)] = \mathbb{E}[\varphi(A,L)I(Y=1)|A=a].$$

Proof. We begin by considering $\mathbb{E}[\varphi^*(A, L)|A = a]$. By the law of the unconscious statistician and Bayes' theorem, we have

$$\begin{aligned}
\mathbb{E}[\varphi^*(A,L)|A=a] &= \sum_l \frac{\Pr(L=l|A=a)}{\Pr(A=a|L=l)} \\
&= \sum_l \frac{\Pr(A=a|L=l)\Pr(L=l)}{\Pr(A=a)\Pr(A=a|L=l)} \\
&= \frac{1}{\Pr(A=a)} \sum_l \Pr(L=l) \\
&= \frac{1}{\Pr(A=a)}.
\end{aligned}$$

Hence, for all a, y, we have

$$\begin{aligned}
\sum_l \varphi(a,l)\Pr(Y=y, L=l|A=a) &= \sum_l \frac{\Pr(Y=y, L=l|A=a)\Pr(A=a)}{\Pr(A=a|L=l)} \\
&= \sum_l \frac{\Pr(Y=y|A=a, L=l)\Pr(A=a|L=l)\Pr(L=l)}{\Pr(A=a|L=l)} \\
&= \sum_l \Pr(Y=y|A=a, L=l)\Pr(L=l) \\
&= \sum_l \Pr(Y(a)=y|A=a, L=l)\Pr(L=l) && \text{(S.1.1)} \\
&= \sum_l \Pr(Y(a)=y|L=l)\Pr(L=l) && \text{(S.1.2)} \\
&= \Pr(Y(a)=y),
\end{aligned}$$

where (S.1.1) and (S.1.2) hold under consistency and conditional exchangeability given L, respectively. Positivity ensures the weights are defined/exist. Hence, $\mathbb{E}[\varphi(A, L)I(Y = 1)|A = a] = \sum_l \varphi(a, l) \Pr(Y = 1, L = l|A = a) = \mathbb{E}[Y(a)]$, as desired.

Corollary A.1. *For any y, a, l, let*

$$\varphi(a,l) = \frac{\varphi^*(a,l)}{\mathbb{E}[\varphi^*(A,L)|A=a]}, \quad \varphi^*(a,l) = \frac{1}{\Pr(A=a|L=l)}, \quad \textit{and}$$
$$\phi(a,l) = \frac{\Pr(Y=1, L=l|A=a)}{\Pr(Z=1, L=l|B=a)}.$$

If $Y(A) = Y$, $(Y(0), Y(1)) \perp\!\!\!\perp A|L$ and positivity holds, then

$$\begin{aligned}
\mathbb{E}[Y(a)] &= \sum_l \varphi(a,l) \Pr(Y=1, L=l|A=a) \\
&= \sum_l \varphi(a,l)\phi(a,l) \Pr(Z=1, L=l|B=a) \\
&= \mathbb{E}[\varphi(B,L)\phi(B,L)I(Z=1)|B=a].
\end{aligned}$$

Appendix II

Theorem S.2. *Fix some $s > 0$ and let $P^* = (Ps+1)/(s+2)$ for all $P \in [0,1]$. If $(P_0, P_1) \in (0,1) \times (0,1)$, then*

$$1 < \frac{P_1^*/(1-P_1^*)}{P_0^*/(1-P_0^*)} < \frac{P_1/(1-P_1)}{P_0/(1-P_0)} \quad \text{if } P_1 > P_0,$$
$$1 = \frac{P_1^*/(1-P_1^*)}{P_0^*/(1-P_0^*)} = \frac{P_1/(1-P_1)}{P_0/(1-P_0)} \quad \text{if } P_1 = P_0, \text{ and}$$
$$1 > \frac{P_1^*/(1-P_1^*)}{P_0^*/(1-P_0^*)} > \frac{P_1/(1-P_1)}{P_0/(1-P_0)} \quad \text{if } P_1 < P_0$$

Proof. Suppose $(P_0, P_1) \in (0,1) \times (0,1)$. If and only if

$$\frac{P_1^*/(1-P_1^*)}{P_0^*/(1-P_0^*)} < \frac{P_1/(1-P_1)}{P_0/(1-P_0)}, \tag{S.2.3}$$

then

$$\frac{P_1 s+1}{s+1-P_1 s}\frac{s+1-P_0 s}{P_0 s+1} < \frac{P_1}{1-P_1}\frac{1-P_0}{P_0},$$
$$\frac{P_1 s+1}{s+1-P_1 s}\frac{1-P_1}{P_1} < \frac{P_0 s+1}{s+1-P_0 s}\frac{1-P_0}{P_0}.$$

Now, since

$$\frac{\partial}{\partial P}\left\{\frac{Ps+1}{s+1-Ps}\frac{1-P}{P}\right\} = \frac{(-2P^2+2P-1)S-1}{P^2(1-(P-1)S)^2} < 0$$

over the interval $(0,1)$ for P, it follows that inequality (S.2.3) holds if $P_1 > P_0$. Also, if $P_1 > P_0$, then, since $\partial/(\partial P)\{(Ps+1)/(s+1-Ps)\} > 0$ if $P \in (0,1)$, we have

$$1 < \frac{P_1^*/(1-P_1^*)}{P_0^*/(1-P_0^*)}.$$

Similar arguments establish the assertion for the case where $P_1 < P_0$. It is easily verified that if $P_1 = P_0$, then

$$\frac{P_1^*/(1-P_1^*)}{P_0^*/(1-P_0^*)} = \frac{P_1 s+1}{s+1-P_1 s}\frac{s+1-P_0 s}{P_0 s+1} = 1$$
$$= \frac{P_1}{1-P_1}\frac{1-P_0}{P_0} = \frac{P_1/(1-P_1)}{P_0/(1-P_0)},$$

as desired.

Appendix III

GP and IPWM were applied to every dataset `data` in R using the function `mecor::ipwm` and the following code:

```
# GP:
formulasGP <- list(
  Y~Z+B+L1+L2+L3+L4+L5+L6+L7+L8+L9+L10,
  B~Z+L1+L2+L3+L4+L5+L6+L7+L8+L9+L10,
  Z~L1+L2+L3+L4+L5+L6+L7+L8+L9+L10
)
mecor::ipwm(
  formulas=formulasGP, data=data, outcome_true=``Y'',
  outcome_mis=``Z'', exposure_true=``B'', exposure_mis=NULL, sp=1e6
)

# IPWM:
formulasIPWM <- list(
  Y~A+Z+B+L1+L2+L3+L4+L5+L6+L7+L8+L9+L10,
  A~Z+B+L1+L2+L3+L4+L5+L6+L7+L8+L9+L10,
  Z~B+L1+L2+L3+L4+L5+L6+L7+L8+L9+L10,
  B~L1+L2+L3+L4+L5+L6+L7+L8+L9+L10
)
mecor::ipwm(
  formulas=formulasIPWM, data=data, outcome_true=``Y'',
  outcome_mis=``Z'', exposure_true=``A'', exposure_mis=``B'', sp=1e6
)
```

Supplementary Tables

Table S.1: Expected cell counts (rounded to integers) for illustrative study setting after misclassification and formation of validation subsets.

					$B=0$		$B=1$	
R_Y	R_A	Y	A	L	$Z=0$	$Z=1$	$Z=0$	$Z=1$
0	0			0	$m_1=9371$	$m_2=7147$	$m_3=1011$	$m_4=884$
0	0			1	$m_5=1120$	$m_6=3165$	$m_7=80$	$m_8=221$
0	1				$m_9=0$	$m_{10}=0$	$m_{11}=0$	$m_{12}=0$
1	0				$m_{13}=0$	$m_{14}=0$	$m_{15}=0$	$m_{16}=0$
1	1	0	0	0	$m_{17}=2728$	$m_{18}=38$	$m_{19}=144$	$m_{20}=2$
1	1	1	0	0	$m_{21}=13$	$m_{22}=3$	$m_{23}=169$	$m_{24}=53$
1	1	0	1	0	$m_{25}=382$	$m_{26}=3797$	$m_{27}=12$	$m_{28}=242$
1	1	1	1	0	$m_{29}=1$	$m_{30}=9$	$m_{31}=12$	$m_{32}=178$
1	1	0	0	1	$m_{33}=287$	$m_{34}=41$	$m_{35}=6$	$m_{36}=5$
1	1	1	0	1	$m_{37}=2$	$m_{38}=1$	$m_{39}=7$	$m_{40}=3$
1	1	0	1	1	$m_{41}=84$	$m_{42}=1658$	$m_{43}=10$	$m_{44}=87$
1	1	1	1	1	$m_{45}=1$	$m_{46}=4$	$m_{47}=3$	$m_{48}=24$

Table S.2: Log-likelihood contributions for all possible types of observations under internal validation sampling.

Type	R_Y	R_A	Z	B	Y	A	L	Count	Log-likelihood contribution
1	0	0	0	0			0	m_1	$\log(1-\varepsilon^*_{00})+\log(1-\delta^*_0)$
2	0	0	1	0			0	m_2	$\log(\varepsilon^*_{00})+\log(1-\delta^*_0)$
3	0	0	0	1			0	m_3	$\log(1-\varepsilon^*_{10})+\log(\delta^*_0)$
4	0	0	1	1			0	m_4	$\log(\varepsilon^*_{10})+\log(\delta^*_0)$
5	0	0	0	0			1	m_5	$\log(1-\varepsilon^*_{01})+\log(1-\delta^*_1)$
6	0	0	1	0			1	m_6	$\log(\varepsilon^*_{01})+\log(1-\delta^*_1)$
7	0	0	0	1			1	m_7	$\log(1-\varepsilon^*_{11})+\log(\delta^*_1)$
8	0	0	1	1			1	m_8	$\log(\varepsilon^*_{11})+\log(\delta^*_1)$
9	0	1						$m_9+\ldots+m_{12}=0$	0
10	1	0						$m_{13}+\ldots+m_{16}=0$	0
11	1	1	0	0	0	0	0	m_{17}	$\log(1-\pi^*_{0000})+\log(1-\lambda^*_{000})+\log(1-\varepsilon^*_{00})+\log(1-\delta^*_0)$
12	1	1	1	0	0	0	0	m_{18}	$\log(1-\pi^*_{0100})+\log(1-\lambda^*_{100})+\log(\varepsilon^*_{00})+\log(1-\delta^*_0)$
13	1	1	0	1	0	0	0	m_{19}	$\log(1-\pi^*_{0010})+\log(1-\lambda^*_{010})+\log(1-\varepsilon^*_{10})+\log(\delta^*_0)$
14	1	1	1	1	0	0	0	m_{20}	$\log(1-\pi^*_{0110})+\log(1-\lambda^*_{110})+\log(\varepsilon^*_{10})+\log(\delta^*_0)$
15	1	1	0	0	1	0	0	m_{21}	$\log(\pi^*_{0000})+\log(1-\lambda^*_{000})+\log(1-\varepsilon^*_{00})+\log(1-\delta^*_0)$
16	1	1	1	0	1	0	0	m_{22}	$\log(\pi^*_{0100})+\log(1-\lambda^*_{100})+\log(\varepsilon^*_{00})+\log(1-\delta^*_0)$
17	1	1	0	1	1	0	0	m_{23}	$\log(\pi^*_{0010})+\log(1-\lambda^*_{010})+\log(1-\varepsilon^*_{10})+\log(\delta^*_0)$
18	1	1	1	1	1	0	0	m_{24}	$\log(\pi^*_{0110})+\log(1-\lambda^*_{110})+\log(\varepsilon^*_{10})+\log(\delta^*_0)$
19	1	1	0	0	0	1	0	m_{25}	$\log(1-\pi^*_{1000})+\log(\lambda^*_{000})+\log(1-\varepsilon^*_{00})+\log(1-\delta^*_0)$
20	1	1	1	0	0	1	0	m_{26}	$\log(1-\pi^*_{1100})+\log(\lambda^*_{100})+\log(\varepsilon^*_{00})+\log(1-\delta^*_0)$
21	1	1	0	1	0	1	0	m_{27}	$\log(1-\pi^*_{1010})+\log(\lambda^*_{010})+\log(1-\varepsilon^*_{10})+\log(\delta^*_0)$
22	1	1	1	1	0	1	0	m_{28}	$\log(1-\pi^*_{1110})+\log(\lambda^*_{110})+\log(\varepsilon^*_{10})+\log(\delta^*_0)$
23	1	1	0	0	1	1	0	m_{29}	$\log(\pi^*_{1000})+\log(\lambda^*_{000})+\log(1-\varepsilon^*_{00})+\log(1-\delta^*_0)$
24	1	1	1	0	1	1	0	m_{30}	$\log(\pi^*_{1100})+\log(\lambda^*_{100})+\log(\varepsilon^*_{00})+\log(1-\delta^*_0)$
25	1	1	0	1	1	1	0	m_{31}	$\log(\pi^*_{1010})+\log(\lambda^*_{010})+\log(1-\varepsilon^*_{10})+\log(\delta^*_0)$
26	1	1	1	1	1	1	0	m_{32}	$\log(\pi^*_{1110})+\log(\lambda^*_{110})+\log(\varepsilon^*_{10})+\log(\delta^*_0)$
27	1	1	0	0	0	0	1	m_{33}	$\log(1-\pi^*_{0001})+\log(1-\lambda^*_{001})+\log(1-\varepsilon^*_{01})+\log(1-\delta^*_1)$
28	1	1	1	0	0	0	1	m_{34}	$\log(1-\pi^*_{0101})+\log(1-\lambda^*_{101})+\log(\varepsilon^*_{01})+\log(1-\delta^*_1)$
29	1	1	0	1	0	0	1	m_{35}	$\log(1-\pi^*_{0011})+\log(1-\lambda^*_{011})+\log(1-\varepsilon^*_{11})+\log(\delta^*_1)$
30	1	1	1	1	0	0	1	m_{36}	$\log(1-\pi^*_{0111})+\log(1-\lambda^*_{111})+\log(\varepsilon^*_{11})+\log(\delta^*_1)$

Table S.2: (continued.)

Type	R_Y	R_A	Z	B	Y	A	L	Count	Log-likelihood contribution
31	1	1	0	0	1	0	1	m_{37}	$\log(\pi^*_{0001}) + \log(1-\lambda^*_{001}) + \log(1-\varepsilon^*_{01}) + \log(1-\delta^*_1)$
32	1	1	1	0	1	0	1	m_{38}	$\log(\pi^*_{0101}) + \log(1-\lambda^*_{101}) + \log(\varepsilon^*_{01}) + \log(1-\delta^*_1)$
33	1	1	0	1	1	0	1	m_{39}	$\log(\pi^*_{0011}) + \log(1-\lambda^*_{011}) + \log(1-\varepsilon^*_{11}) + \log(\delta^*_1)$
34	1	1	1	1	1	0	1	m_{40}	$\log(\pi^*_{0111}) + \log(1-\lambda^*_{111}) + \log(\varepsilon^*_{11}) + \log(\delta^*_1)$
35	1	1	0	0	0	1	1	m_{41}	$\log(1-\pi^*_{1001}) + \log(\lambda^*_{001}) + \log(1-\varepsilon^*_{01}) + \log(1-\delta^*_1)$
36	1	1	1	0	0	1	1	m_{42}	$\log(1-\pi^*_{1101}) + \log(\lambda^*_{101}) + \log(\varepsilon^*_{01}) + \log(1-\delta^*_1)$
37	1	1	0	1	0	1	1	m_{43}	$\log(1-\pi^*_{1011}) + \log(\lambda^*_{011}) + \log(1-\varepsilon^*_{11}) + \log(\delta^*_1)$
38	1	1	1	1	0	1	1	m_{44}	$\log(1-\pi^*_{1111}) + \log(\lambda^*_{111}) + \log(\varepsilon^*_{11}) + \log(\delta^*_1)$
39	1	1	0	0	1	1	1	m_{45}	$\log(\pi^*_{1001}) + \log(\lambda^*_{001}) + \log(1-\varepsilon^*_{01}) + \log(1-\delta^*_1)$
40	1	1	1	0	1	1	1	m_{46}	$\log(\pi^*_{1101}) + \log(\lambda^*_{101}) + \log(\varepsilon^*_{01}) + \log(1-\delta^*_1)$
41	1	1	0	1	1	1	1	m_{47}	$\log(\pi^*_{1011}) + \log(\lambda^*_{011}) + \log(1-\varepsilon^*_{11}) + \log(\delta^*_1)$
42	1	1	1	1	1	1	1	m_{48}	$\log(\pi^*_{1111}) + \log(\lambda^*_{111}) + \log(\varepsilon^*_{11}) + \log(\delta^*_1)$

Table S.3: Closed form expressions for the maximum likelihood estimators (MLE) of the parameters of the likelihood parameterised in terms of predictive values for the hypothetical study setting.

Parameter	MLE
δ_0^*	$\hat{\delta}_0^* = (m_3 + m_4 + m_{19} + m_{20} + m_{23} + m_{24} + m_{27} + m_{28} + m_{31} + m_{32})/(\{\sum_{j=1}^{4} m_j\} + \{\sum_{j=17}^{32} m_j\})$
δ_1^*	$\hat{\delta}_1^* = (m_7 + m_8 + m_{35} + m_{36} + m_{39} + m_{40} + m_{43} + m_{44} + m_{47} + m_{48})/(\{\sum_{j=5}^{8} m_j\} + \{\sum_{j=33}^{48} m_j\})$
ε_{00}^*	$\hat{\varepsilon}_{00}^* = (m_2 + m_{18} + m_{22} + m_{26} + m_{30})/(m_1 + m_2 + m_{17} + m_{18} + m_{21} + m_{22} + m_{25} + m_{26} + m_{29} + m_{30})$
ε_{10}^*	$\hat{\varepsilon}_{10}^* = (m_4 + m_{20} + m_{24} + m_{28} + m_{32})/(m_3 + m_4 + m_{19} + m_{20} + m_{23} + m_{24} + m_{27} + m_{28} + m_{31} + m_{32})$
ε_{01}^*	$\hat{\varepsilon}_{01}^* = (m_6 + m_{34} + m_{38} + m_{42} + m_{46})/(m_5 + m_6 + m_{33} + m_{34} + m_{37} + m_{38} + m_{41} + m_{42} + m_{45} + m_{46})$
ε_{11}^*	$\hat{\varepsilon}_{11}^* = (m_8 + m_{36} + m_{40} + m_{44} + m_{48})/(m_7 + m_8 + m_{35} + m_{36} + m_{39} + m_{40} + m_{43} + m_{44} + m_{47} + m_{48})$
λ_{000}^*	$\hat{\lambda}_{000}^* = (m_{25} + m_{29})/(m_{17} + m_{21} + m_{25} + m_{29})$
λ_{100}^*	$\hat{\lambda}_{100}^* = (m_{26} + m_{30})/(m_{18} + m_{22} + m_{26} + m_{30})$
λ_{010}^*	$\hat{\lambda}_{010}^* = (m_{27} + m_{31})/(m_{19} + m_{23} + m_{27} + m_{31})$
λ_{110}^*	$\hat{\lambda}_{110}^* = (m_{28} + m_{32})/(m_{20} + m_{24} + m_{28} + m_{32})$
λ_{001}^*	$\hat{\lambda}_{001}^* = (m_{41} + m_{45})/(m_{33} + m_{37} + m_{41} + m_{45})$
λ_{101}^*	$\hat{\lambda}_{101}^* = (m_{42} + m_{46})/(m_{34} + m_{38} + m_{42} + m_{46})$
λ_{011}^*	$\hat{\lambda}_{011}^* = (m_{43} + m_{47})/(m_{35} + m_{39} + m_{43} + m_{47})$
λ_{111}^*	$\hat{\lambda}_{111}^* = (m_{44} + m_{48})/(m_{36} + m_{40} + m_{44} + m_{48})$
π_{0000}^*	$\hat{\pi}_{0000}^* = m_{21}/(m_{17} + m_{21})$
π_{1000}^*	$\hat{\pi}_{1000}^* = m_{29}/(m_{25} + m_{29})$
π_{0100}^*	$\hat{\pi}_{0100}^* = m_{22}/(m_{18} + m_{22})$
π_{1100}^*	$\hat{\pi}_{1100}^* = m_{30}/(m_{26} + m_{30})$
π_{0010}^*	$\hat{\pi}_{0010}^* = m_{23}/(m_{19} + m_{23})$
π_{1010}^*	$\hat{\pi}_{1010}^* = m_{31}/(m_{27} + m_{31})$
π_{0110}^*	$\hat{\pi}_{0110}^* = m_{24}/(m_{20} + m_{24})$
π_{1110}^*	$\hat{\pi}_{1110}^* = m_{32}/(m_{28} + m_{32})$
π_{0001}^*	$\hat{\pi}_{0001}^* = m_{37}/(m_{33} + m_{37})$
π_{1001}^*	$\hat{\pi}_{1001}^* = m_{45}/(m_{41} + m_{45})$
π_{0101}^*	$\hat{\pi}_{0101}^* = m_{38}/(m_{34} + m_{38})$
π_{1101}^*	$\hat{\pi}_{1101}^* = m_{46}/(m_{42} + m_{46})$
π_{0011}^*	$\hat{\pi}_{0011}^* = m_{39}/(m_{35} + m_{39})$
π_{1011}^*	$\hat{\pi}_{1011}^* = m_{47}/(m_{43} + m_{47})$
π_{0111}^*	$\hat{\pi}_{0111}^* = m_{40}/(m_{36} + m_{40})$
π_{1111}^*	$\hat{\pi}_{1111}^* = m_{48}/(m_{44} + m_{48})$

Table S.4: Results for simulation studies 1a-18a,1b-18b,1c-18c on the performance of different causal estimators in various scenarios of confounding and misclassification in exposure and outcome. Abbreviations: PS, propensity score method ignoring misclassification; CCA, complete case analysis; GP, Gravel and Platt estimator ignoring exposure misclassification; IPWM, inverse probability weighting method for confounding and joint exposure and outcome misclassification; ... (continued on next page).

	Crude						PS					
Scenario	Bias	BSE	MSE	SE	SSE	CP	Bias	BSE	MSE	SE	SSE	CP
1a	0.401	0.003	0.167	0.081	0.083	0.004	0.399	0.004	0.173	0.117	0.120	0.080
2a	0.392	0.004	0.170	0.127	0.127	0.189	0.391	0.006	0.184	0.177	0.181	0.436
3a	0.400	0.003	0.167	0.083	0.083	0.005	0.391	0.004	0.167	0.119	0.119	0.104
4a	0.394	0.003	0.162	0.081	0.083	0.007	0.392	0.004	0.169	0.122	0.120	0.106
5a	0.398	0.002	0.162	0.061	0.062	0.000	0.398	0.002	0.162	0.061	0.062	0.000
6a	0.404	0.003	0.172	0.094	0.094	0.010	0.404	0.003	0.172	0.094	0.095	0.011
7a	0.399	0.002	0.163	0.062	0.062	0.000	0.399	0.002	0.163	0.062	0.062	0.000
8a	0.401	0.002	0.165	0.064	0.062	0.000	0.401	0.002	0.165	0.064	0.062	0.000
9a	0.400	0.002	0.164	0.064	0.062	0.000	0.400	0.002	0.164	0.064	0.062	0.000
10a	0.396	0.003	0.164	0.085	0.083	0.004	0.395	0.004	0.171	0.123	0.119	0.101
11a	0.396	0.004	0.173	0.128	0.127	0.176	0.388	0.006	0.185	0.187	0.182	0.455
12a	0.398	0.003	0.165	0.081	0.083	0.007	0.398	0.004	0.173	0.120	0.120	0.096
13a	0.399	0.003	0.166	0.083	0.083	0.004	0.395	0.004	0.171	0.120	0.119	0.102
14a	0.404	0.002	0.167	0.061	0.062	0.000	0.404	0.002	0.167	0.061	0.062	0.000
15a	0.398	0.003	0.167	0.092	0.094	0.011	0.398	0.003	0.167	0.092	0.095	0.012
16a	0.404	0.002	0.167	0.063	0.062	0.000	0.404	0.002	0.167	0.063	0.062	0.000
17a	0.399	0.002	0.163	0.061	0.062	0.000	0.399	0.002	0.163	0.061	0.062	0.000
18a	0.401	0.002	0.164	0.059	0.062	0.000	0.401	0.002	0.164	0.059	0.062	0.000
1b	0.394	0.004	0.169	0.119	0.118	0.122	0.392	0.005	0.182	0.168	0.169	0.382
2b	0.382	0.006	0.179	0.183	0.184	0.492	0.379	0.008	0.213	0.264	0.258	0.738
3b	0.394	0.004	0.169	0.117	0.118	0.116	0.389	0.006	0.182	0.175	0.169	0.402
4b	0.401	0.004	0.174	0.117	0.118	0.102	0.389	0.006	0.182	0.176	0.168	0.392
5b	0.401	0.003	0.169	0.090	0.088	0.007	0.402	0.003	0.170	0.090	0.088	0.010
6b	0.407	0.004	0.183	0.132	0.134	0.133	0.407	0.004	0.183	0.131	0.135	0.136
7b	0.396	0.003	0.164	0.086	0.088	0.009	0.396	0.003	0.164	0.086	0.088	0.009
8b	0.395	0.003	0.164	0.086	0.088	0.005	0.395	0.003	0.164	0.086	0.088	0.004
9b	0.398	0.003	0.166	0.089	0.088	0.005	0.398	0.003	0.166	0.089	0.088	0.005
10b	0.397	0.004	0.171	0.117	0.118	0.100	0.396	0.005	0.185	0.167	0.170	0.387
11b	0.391	0.006	0.185	0.179	0.183	0.466	0.362	0.008	0.199	0.261	0.253	0.732
12b	0.401	0.004	0.174	0.118	0.118	0.109	0.391	0.005	0.182	0.173	0.169	0.394
13b	0.404	0.004	0.176	0.111	0.117	0.080	0.396	0.005	0.185	0.169	0.167	0.367
14b	0.400	0.003	0.168	0.087	0.088	0.008	0.400	0.003	0.168	0.087	0.088	0.006
15b	0.397	0.004	0.176	0.135	0.134	0.161	0.397	0.004	0.176	0.135	0.135	0.161
16b	0.401	0.003	0.168	0.087	0.088	0.006	0.400	0.003	0.168	0.087	0.088	0.006
17b	0.403	0.003	0.170	0.087	0.088	0.003	0.403	0.003	0.170	0.087	0.088	0.004
18b	0.400	0.003	0.168	0.087	0.088	0.004	0.400	0.003	0.168	0.088	0.088	0.003
1c	0.394	0.009	0.232	0.277	0.275	0.698	0.366	0.013	0.292	0.398	0.391	0.871
2c	0.334	0.018	0.423	0.558	0.844	0.873	0.256	0.022	0.563	0.706	0.924	0.916
3c	0.383	0.009	0.222	0.274	0.276	0.739	0.371	0.013	0.297	0.399	0.393	0.875
4c	0.375	0.009	0.218	0.278	0.277	0.732	0.332	0.013	0.276	0.407	0.392	0.880
5c	0.405	0.006	0.204	0.200	0.199	0.470	0.405	0.006	0.205	0.201	0.199	0.474
6c	0.410	0.010	0.261	0.304	0.317	0.724	0.410	0.010	0.263	0.308	0.318	0.729
7c	0.406	0.006	0.203	0.196	0.199	0.469	0.406	0.006	0.204	0.198	0.200	0.469
8c	0.404	0.006	0.204	0.202	0.199	0.474	0.405	0.006	0.205	0.201	0.200	0.470
9c	0.406	0.006	0.202	0.192	0.198	0.468	0.404	0.006	0.201	0.193	0.199	0.470
10c	0.384	0.009	0.222	0.272	0.276	0.717	0.359	0.013	0.288	0.399	0.388	0.873
11c	0.358	0.014	0.324	0.443	0.825	0.864	0.296	0.020	0.471	0.619	0.902	0.923
12c	0.377	0.008	0.212	0.265	0.277	0.749	0.343	0.013	0.284	0.407	0.393	0.878
13c	0.377	0.008	0.210	0.259	0.276	0.741	0.341	0.013	0.274	0.397	0.390	0.888
14c	0.411	0.006	0.206	0.192	0.199	0.446	0.411	0.006	0.206	0.192	0.200	0.458
15c	0.393	0.009	0.241	0.294	0.315	0.764	0.393	0.009	0.242	0.296	0.316	0.770
16c	0.399	0.006	0.198	0.196	0.198	0.484	0.399	0.006	0.198	0.196	0.200	0.482
17c	0.395	0.006	0.193	0.191	0.199	0.471	0.394	0.006	0.191	0.190	0.199	0.474
18c	0.402	0.006	0.201	0.197	0.199	0.478	0.403	0.006	0.202	0.199	0.200	0.482

Table S.4: (Continued.) ... BSE, estimated standard error for the bias due to Monte Carlo error; SE, empirical standard error; SSE, sample standard error; CP, empirical coverage probability. In all scenarios, the true marginal log OR (estimand) was -0.4

	CCA						GP					
Scenario	Bias	BSE	MSE	SE	SSE	CP	Bias	BSE	MSE	SE	SSE	CP
1a	−0.011	0.010	0.091	0.302	0.315	0.932	−0.016	0.008	0.059	0.242	0.257	0.960
2a	−0.038	0.013	0.165	0.404	0.398	0.909	−0.024	0.010	0.108	0.328	0.353	0.956
3a	0.004	0.007	0.044	0.210	0.208	0.939	−0.013	0.005	0.028	0.167	0.165	0.943
4a	−0.050	0.014	0.189	0.432	0.441	0.905	−0.022	0.011	0.116	0.341	0.371	0.944
5a	−0.128	0.006	0.054	0.194	0.199	0.890	0.271	0.005	0.100	0.163	0.168	0.633
6a	−0.097	0.007	0.066	0.237	0.245	0.926	0.269	0.007	0.121	0.221	0.225	0.772
7a	−0.232	0.005	0.082	0.168	0.173	0.736	0.118	0.005	0.043	0.171	0.173	0.904
8a	−0.197	0.004	0.056	0.130	0.130	0.646	0.263	0.003	0.079	0.098	0.101	0.261
9a	−0.173	0.008	0.101	0.266	0.270	0.883	0.257	0.007	0.116	0.224	0.229	0.795
10a	0.017	0.005	0.029	0.169	0.170	0.953	0.003	0.005	0.022	0.147	0.152	0.946
11a	0.007	0.006	0.040	0.200	0.193	0.947	−0.014	0.006	0.039	0.196	0.203	0.952
12a	0.058	0.005	0.028	0.157	0.154	0.928	−0.003	0.004	0.019	0.138	0.136	0.943
13a	0.018	0.007	0.056	0.236	0.237	0.946	−0.003	0.006	0.037	0.192	0.194	0.940
14a	−0.092	0.003	0.018	0.099	0.105	0.864	0.265	0.003	0.079	0.091	0.095	0.191
15a	−0.051	0.004	0.016	0.115	0.119	0.933	0.264	0.004	0.084	0.121	0.124	0.421
16a	−0.166	0.003	0.036	0.094	0.092	0.559	0.138	0.003	0.028	0.096	0.096	0.710
17a	−0.116	0.003	0.023	0.095	0.094	0.762	0.264	0.003	0.076	0.080	0.082	0.110
18a	−0.115	0.005	0.035	0.149	0.144	0.859	0.266	0.004	0.088	0.131	0.128	0.455
1b	−0.078	0.015	0.226	0.469	0.491	0.899	−0.036	0.011	0.130	0.359	0.428	0.958
2b	−0.117	0.019	0.375	0.601	0.900	0.887	−0.097	0.016	0.265	0.505	0.861	0.938
3b	−0.020	0.010	0.091	0.301	0.300	0.919	−0.019	0.007	0.055	0.233	0.240	0.939
4b	−0.093	0.020	0.407	0.631	1.158	0.899	−0.045	0.016	0.253	0.501	1.087	0.944
5b	−0.145	0.009	0.103	0.286	0.286	0.903	0.269	0.008	0.132	0.244	0.244	0.799
6b	−0.109	0.011	0.131	0.345	0.362	0.930	0.280	0.010	0.177	0.314	0.339	0.862
7b	−0.213	0.007	0.101	0.237	0.250	0.865	0.134	0.008	0.076	0.241	0.252	0.926
8b	−0.209	0.006	0.079	0.187	0.186	0.775	0.259	0.004	0.087	0.140	0.144	0.570
9b	−0.175	0.012	0.184	0.392	0.411	0.902	0.263	0.010	0.174	0.325	0.339	0.883
10b	0.011	0.007	0.056	0.237	0.244	0.957	−0.002	0.007	0.050	0.223	0.221	0.939
11b	0.001	0.009	0.083	0.288	0.276	0.918	−0.019	0.010	0.093	0.304	0.304	0.938
12b	0.058	0.007	0.050	0.216	0.223	0.953	−0.007	0.006	0.038	0.194	0.197	0.949
13b	−0.015	0.011	0.122	0.350	0.345	0.934	−0.023	0.009	0.077	0.277	0.287	0.950
14b	−0.092	0.005	0.030	0.146	0.148	0.889	0.263	0.004	0.088	0.136	0.136	0.505
15b	−0.060	0.005	0.033	0.170	0.170	0.929	0.263	0.006	0.101	0.177	0.183	0.712
16b	−0.171	0.004	0.047	0.132	0.131	0.741	0.139	0.004	0.038	0.136	0.138	0.820
17b	−0.121	0.004	0.032	0.134	0.135	0.842	0.263	0.004	0.082	0.115	0.118	0.388
18b	−0.113	0.007	0.055	0.206	0.207	0.904	0.264	0.006	0.102	0.178	0.185	0.702
1c	−1.163	0.098	10.972	3.101	3.092	0.792	−0.994	0.095	10.003	3.003	3.085	0.878
2c	−2.086	0.131	21.614	4.155	3.415	0.733	−1.979	0.130	20.875	4.118	3.689	0.835
3c	−0.184	0.029	0.880	0.920	1.477	0.887	−0.102	0.024	0.574	0.751	1.412	0.939
4c	−2.730	0.148	29.275	4.671	3.710	0.722	−2.295	0.142	25.436	4.491	3.974	0.916
5c	−0.254	0.029	0.904	0.916	1.764	0.891	0.288	0.018	0.409	0.571	1.106	0.951
6c	−0.548	0.067	4.832	2.129	2.684	0.893	0.402	0.046	2.276	1.454	2.782	0.969
7c	−0.223	0.020	0.467	0.646	1.303	0.912	0.109	0.020	0.424	0.642	1.322	0.952
8c	−0.236	0.014	0.258	0.450	0.508	0.891	0.279	0.011	0.197	0.345	0.369	0.883
9c	−0.662	0.079	6.624	2.487	3.186	0.896	0.314	0.053	2.915	1.678	2.454	0.972
10c	−0.105	0.019	0.376	0.604	0.740	0.897	−0.098	0.017	0.294	0.534	0.790	0.943
11c	−0.113	0.025	0.649	0.798	1.174	0.906	−0.183	0.031	1.006	0.986	1.758	0.931
12c	0.010	0.018	0.313	0.560	0.598	0.938	−0.053	0.021	0.455	0.673	0.703	0.937
13c	−0.170	0.036	1.330	1.140	1.967	0.919	−0.129	0.030	0.920	0.950	1.983	0.948
14c	−0.107	0.011	0.133	0.349	0.360	0.922	0.286	0.010	0.187	0.324	0.351	0.882
15c	−0.088	0.013	0.187	0.424	0.417	0.913	0.276	0.015	0.314	0.488	0.664	0.951
16c	−0.159	0.009	0.115	0.299	0.311	0.924	0.143	0.010	0.124	0.323	0.357	0.952
17c	−0.133	0.010	0.114	0.311	0.323	0.918	0.259	0.009	0.144	0.277	0.304	0.872
18c	−0.148	0.016	0.264	0.492	0.593	0.938	0.280	0.014	0.278	0.447	0.510	0.926

Table S.4: (Continued.)

Scenario	IPWM					
	Bias	BSE	MSE	SE	SSE	CP
1a	−0.016	0.008	0.059	0.242	0.257	0.960
2a	−0.024	0.010	0.108	0.328	0.353	0.956
3a	−0.013	0.005	0.028	0.167	0.165	0.943
4a	−0.022	0.011	0.116	0.341	0.371	0.944
5a	−0.004	0.006	0.035	0.186	0.194	0.954
6a	−0.010	0.008	0.060	0.244	0.252	0.952
7a	−0.013	0.005	0.030	0.172	0.179	0.951
8a	0.003	0.004	0.015	0.122	0.125	0.952
9a	−0.019	0.008	0.065	0.255	0.265	0.948
10a	0.003	0.005	0.022	0.147	0.152	0.946
11a	−0.014	0.006	0.039	0.196	0.203	0.952
12a	−0.003	0.004	0.019	0.138	0.136	0.943
13a	−0.003	0.006	0.037	0.192	0.194	0.940
14a	−0.005	0.003	0.011	0.104	0.106	0.962
15a	−0.001	0.004	0.017	0.129	0.134	0.963
16a	0.010	0.003	0.010	0.099	0.099	0.947
17a	0.001	0.003	0.009	0.096	0.095	0.943
18a	0.001	0.005	0.022	0.148	0.144	0.949
1b	−0.036	0.011	0.130	0.359	0.428	0.958
2b	−0.097	0.016	0.265	0.505	0.861	0.938
3b	−0.019	0.007	0.055	0.233	0.240	0.939
4b	−0.045	0.016	0.253	0.501	1.087	0.944
5b	−0.017	0.009	0.082	0.286	0.284	0.942
6b	−0.014	0.011	0.129	0.359	0.386	0.958
7b	0.004	0.008	0.059	0.243	0.261	0.969
8b	−0.004	0.006	0.032	0.180	0.181	0.958
9b	−0.025	0.012	0.141	0.374	0.415	0.956
10b	−0.002	0.007	0.050	0.223	0.221	0.939
11b	−0.019	0.010	0.093	0.304	0.304	0.938
12b	−0.007	0.006	0.038	0.194	0.197	0.949
13b	−0.023	0.009	0.077	0.277	0.287	0.950
14b	−0.003	0.005	0.022	0.147	0.152	0.960
15b	−0.006	0.006	0.035	0.187	0.198	0.963
16b	0.010	0.004	0.020	0.142	0.143	0.956
17b	−0.003	0.004	0.017	0.131	0.136	0.956
18b	0.010	0.006	0.042	0.205	0.207	0.955
1c	−0.994	0.095	10.003	3.003	3.085	0.878
2c	−1.979	0.130	20.875	4.118	3.689	0.835
3c	−0.102	0.024	0.574	0.751	1.412	0.939
4c	−2.295	0.142	25.436	4.491	3.974	0.916
5c	−0.101	0.029	0.849	0.916	1.771	0.950
6c	−0.373	0.069	4.896	2.181	3.041	0.978
7c	−0.027	0.022	0.470	0.685	1.298	0.961
8c	−0.019	0.014	0.200	0.447	0.527	0.953
9c	−0.372	0.068	4.769	2.152	2.579	0.989
10c	−0.098	0.017	0.294	0.534	0.790	0.943
11c	−0.183	0.031	1.006	0.986	1.758	0.931
12c	−0.053	0.021	0.455	0.673	0.703	0.937
13c	−0.129	0.030	0.920	0.950	1.983	0.948
14c	−0.003	0.011	0.130	0.360	0.396	0.967
15c	−0.018	0.016	0.263	0.512	0.705	0.984
16c	0.014	0.011	0.114	0.338	0.371	0.966
17c	−0.005	0.010	0.101	0.318	0.349	0.952
18c	−0.002	0.016	0.249	0.499	0.613	0.962
